# Anchoring of heterologous proteins in multiple *Lactobacillus* species using anchors derived from *Lactobacillus plantarum*

**DOI:** 10.1038/s41598-020-66531-7

**Published:** 2020-06-15

**Authors:** Geir Mathiesen, Lise Øverland, Katarzyna Kuczkowska, Vincent G. H. Eijsink

**Affiliations:** Faculty of Chemistry, Biotechnology and Food Science, NMBU - Norwegian University of Life Sciences, Ås, Norway

**Keywords:** Biotechnology, Microbiology, Molecular biology

## Abstract

Members of the genus *Lactobacillus* have a long history in food applications and are considered as promising and safe hosts for delivery of medically interesting proteins. We have assessed multiple surface anchors derived from *Lactobacillus plantarum* for protein surface display in multiple *Lactobacillus* species, using a *Mycobacterium tuberculosis* hybrid antigen as test protein. The anchors tested were a lipoprotein anchor and two cell wall anchors, one non-covalent (LysM domain) and one covalent (sortase-based anchoring using the LPXTG motif). Thus, three different expression vectors for surface-anchoring were tested in eight *Lactobacillus* species. When using the LPXTG and LysM cell wall anchors, surface display, as assessed by flow cytometry and fluorescence microscopy, was observed in all species except *Lactobacillus acidophilus*. Use of the cell membrane anchor revealed more variation in the apparent degree of surface-exposure among the various lactobacilli. Overproduction of the secreted and anchored antigen impaired bacterial growth rate to extents that varied among the lactobacilli and were dependent on the type of anchor. Overall, these results show that surface anchors derived from *L*. *plantarum* are promising candidates for efficient anchoring of medically interesting proteins in other food grade *Lactobacillus* species.

## Introduction

The genus *Lactobacillus* consists of more than 200 species with substantial economic importance due to use in food products and in biotechnological and therapeutic applications^1^. Lactobacilli have a long history of safe use in humans. They occur in many food products and have the GRAS (Generally Recognized As Safe) status, and several lactobacilli of human origin are commercialized under brand names^2^.

**Figure 1:**
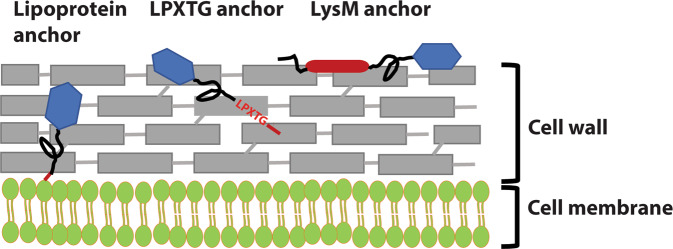
Schematic overview over the anchors. The red color indicates the various anchoring domains and motifs, whereas the black color indicates the linker regions between the anchor and the fused antigen, in blue.

Because lactobacilli are safe and may have immune-stimulating adjuvant effects^3–8^, they are promising delivery vectors for antigens and other medical molecules. Studies with animal models have repeatedly demonstrated the potential of antigen producing lactobacilli to induce specific immune responses^9–16^ and one such *Lactobacillus* has even reached clinical tests^17^. Ideally, the antigens should be sufficiently protected from proteolytic digestion and other damage in the harsh environment of the gastro-intestinal tract, while at the same time being sufficiently exposed to provoke favorable immune responses at mucosal surfaces. Secreted and released antigens will easily be damaged, whereas antigens embedded in the cell wall may be more protected but also less accessible for the immune system. Therefore, when creating the expression system, careful consideration of the subcellular location of the antigen is of importance, since different localization at the bacterial surface will result in different responses^18,19^. Figure 1 illustrates that key strategies for anchoring vary in terms of the expected degree of exposure of the antigen on the bacterial surface^20^.

One strategy for surface-anchoring is to utilize lipoproteins, which contain an N-terminal signal sequence with a signal peptidase (SPase II) cleavage site. Secretion and SPase II-mediated cleavage is accompanied by coupling a lipid to the N-terminal cysteine residue of the SPase II-cleaved protein and the lipid moiety keeps the protein associated to the membrane^21^. Fusing the N-terminus of a target protein to the N-terminal part of a natural lipoprotein, downstream of the conserved cysteine, may thus lead to covalent anchoring to the cell membrane. Only a few studies have shown successful anchoring and surface display using lipoprotein anchors in *Lactobacillus*^20^.

Targeting heterologous proteins covalently to the cell wall can be done by fusing the target protein to the C-terminal part of proteins containing the so-called LPXTG motif ^22^. In natural surface-displayed proteins the LPXTG motif is followed by a hydrophobic trans-membrane sequence and a cluster of positively charged amino acids. During translocation, a cell membrane located sortase enzyme cleaves between the threonine and glycine, while covalently attaching the threonine residue to the peptidoglycan layer^23^. Several studies have shown successful LPXTG-based covalent anchoring of a target protein to the cell wall of lactobacilli^11,19,24–27^.

Non-covalent targeting to the cell wall is also an option and can be achieved by attaching peptidoglycan-binding LysM domains to the protein of interest. Such domains are present in single or multiple copies in natural proteins and bind specifically to *N*-acetylglucosamine moieties in the cell wall. Addition of LysM domains has been widely used for surface-targeting of proteins, as reviewed in^28^. Of-note, LysM domains can be exploited in non-GMO strategies for surface display. In such a strategy, one would produce the fusion protein with a genetically modified producer organism and the purified fusion protein can then in principle be used to decorate the surface of any (non-GMO) bacterium that contains peptidoglycan, such as natural *Lactobacillus* species.

It has previously been shown that the use of various anchor types, which likely lead to varying locations of the displayed protein, affect the downstream responses^9,18^. Different species of *Lactobacillus* have different surface structures^29^, which may affect surface exposure of the anchored protein as well as immune-modulatory effects^8^. In addition, *Lactobacillus* species may vary in terms of the amount of antigen that they manage to display, which also can affect downstream responses. For example, a previous study in which a lipoprotein-anchored tuberculosis antigen (AgE6) was expressed in *L*. *plantarum, L*. *brevis, L*. *gasseri* and *L*. *reuteri* showed that the resulting recombinant strains gave clearly different immune responses in mice^30^. It was also shown, *in vitro*, that these four species were able to activate human dendritic cells (DCs), which is crucial in T-cell responses to vaccines. Thus, in the search for developing potential *Lactobacillus-*based vaccines, it is worth looking at different surface anchors as well as different *Lactobacillus* species.

In the present study, we evaluated the potential of using three different surface anchors derived from *Lactobacillus plantarum* for targeting a *Mycobacterium tuberculosis* hybrid antigen in eight different species of *Lactobacillus*: *L*. *plantarum, L*. *gasseri, L*. *reuteri, L*. *acidophilus, L*. *sakei, L*. *rhamnosus, L*. *curvatus* and *L*. *brevis*. The strains used included both human and food isolates and the species represent different phylogenetic groups within the genus *Lactobacillus*. To assess various anchors, we have compared the functionality of a previously studied lipoprotein anchor^30^ and two cell wall binding anchors (LPXTG and LysM) using Western blotting, to assess protein production, as well as flow cytometry and immunofluorescence microscopy, to asses surface localization. Generally, this study showed that most constructs for anchoring did result in surface-display of the antigen, highlighting the potential of the tested *Lactobacillus* species as delivery vectors for medically interesting proteins.

## Results and discussion

We have previously constructed vectors for inducible intracellular production of heterologous proteins, the so-called pSIP vectors^31,32^. These vectors have been further developed for secretion^33^ and surface display of proteins of interest in *L*. *plantarum*^18,24^. In these previous studies, the secretion and anchoring vectors contained the narrow host range 256_rep_ replicon^34^, which limits the use of the constructs to very few species of *Lactobacillus*. To expand the host strains we recently developed vectors with a broad range replicon that enabled propagation of pSIP vectors in all eight *Lactobacillus* species used in the present study (Table 1) and allowed pSIP-based secretion of heterologous proteins in most of these^35^. This latter study showed that signal peptides derived from *L*. *plantarum* could be used for secretion of nuclease A (NucA) in five different lactobacilli. To deliver proteins to mucosal layers, it may be more beneficial to display the protein on the bacterial surface, since the proteins are more exposed while possibly being protected from harsh conditions by the confinement of the cell wall.

**Table 1:** Bacterial strains and plasmid used in this study.

Strains	Comments, origin	References or source
*Lactococcus lactis* IL1403	Subcloning host strain	^47^
*Lactobacillus plantarum* WCFS1	Human saliva, secretion host	^44^
*L*. *brevis* DSM20556	Green olives, secretion host	DSMZ
*L*. *rhamnosus* GG	Human GI tract, secretion host	Valio Ltd, Finland^48^
*L*. *curvatus* DSM 20019	Milk, secretion host	DSMZ
*L*. *gasseri* ATCC 33323	Human GI tract, secretion host	^49^
*L*. *sakei* Lb790	Meat, secretion host	^50^
*L*. *reuteri* DSM 20016	Human GI tract, secretion host	DSMZ
*L*. *acidophilus* ATCC 4356	Human GI tract, secretion host	ATCC
**Plasmids**	**Relevant characteristics**	**References or source**
pEV	pSIP401^31^ derivative without target gene, “empty vector”; Em^R^	^18^
pUC57_AgE6	Amp^R^, pUC57 vector with synthetic gene encoding Ag85B-ESAT-6 (AgE6)	Genscript, Piscataway, NJ
pLp_0373sNucA	pSIP401 derivative with *nucA* fused to signal peptide *Lp_0373*; 256_rep_. Em^R^	^51^
pLp3014Inv	pSIP401 derivative, encoding Invasin fused to a signal peptide and N-terminal LysM anchor derived from *Lp_3014;* 256_rep;_ Em^R^	^18^
pCyt (pLp_cyt:AgE6-DC_SH71)	pSIP401 derivative for intracellular production of the Ag85B_ESAT-6 (AgE6) hybrid protein fused to a DC-binding sequence; SH71_rep_; Em^R^	This study
pLp_1261AgE6-DC	pSIP401 derivative, encoding a lipoprotein anchor sequence derived from *Lp_1261* fused to the AgE6-DC hybrid protein; 256_rep_; Em^R^	^9^
pLipo (pLp_1261AE6-DC_SH71)	pSIP401 derivative, encoding a lipoprotein anchor sequence derived from *Lp_1261* fused to the AgE6-DC hybrid protein; SH71_rep_; Em^R^	^30^
pCwa2 (pLp_3050DC_AgE6cwa2_SH71)	pSIP401 derivative, encoding signal peptide *Lp_3050* fused to DC binding sequence followed by the AgE6 hybrid protein and a subsequent LPXTG anchor sequence (Cwa2); SH71_rep_; Em^R^	This study
pLysM (pLp_3014_AgE6-DC_SH71)	pSIP401 derivative, encoding a signal peptide followed by a LysM domain, derived from *Lp_3014*, fused to AgE6-DC	This study

The three anchoring sequences used in the present study are derived from *L*. *plantarum* and have previously successfully used for surface display of invasin, an antibody and a HIV antigen on the surface of *L*. *plantarum*^18,36,37^. In a previous study, we have translationally fused the lipoprotein anchor to a *M*. *tuberculosis* fusion antigen (Ag85B & ESAT-6, referred to as AgE6) and inserted the resulting gene into a pSIP derivative containing the SH71 replicon, allowing vector propagation and assessment of antigen production and localization in multiple lactobacilli^30^. Here, we constructed three more vectors, pCwa2 (LPXTG cell wall anchor) and pLysM (LysM cell wall anchor), and a control plasmid for intracellular production of AgE6 (pCyt). In accordance with previous experiences with the vector for lipoprotein anchoring (pLipo) viable transformants for all eight tested lactobacilli were obtained for all three novel vectors.

**Figure 2:**
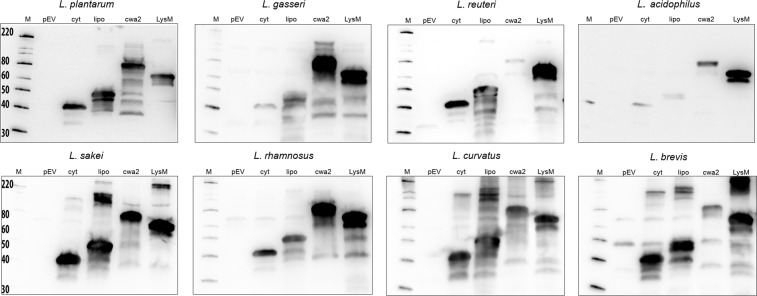
Production of the antigen. The pictures show western blots of cell-free extracts ofAg85B-ESAT6-DC(AgE6-DC) expressing strains harvested 3 hours after induction. Sample sizes were adjusted to the OD_600_ of the harvested culture, meaning that all samples represent approximately equal amounts of cells. Lanes: M, molecular mass markers (masses are indicated in kDa); pEV, strain harboring empty vector; cyt, strain harboring vector for intracellular localization (expected mass of the fusion protein is 41 kDa); lipo (48 kDa), cwa2 (69 kDa) and LysM (66 kDa), cell-free extracts of strains harboring various plasmids for anchoring (expected masses between parenthesis). The data presented are from one representative experiment, out of at least three experiments in total. Parts of the lanes marked “lipo” have been published previously^30^, except in the case of *L*. *sakei*.

To examine the production of the AgE6 antigen, the cells were induced by 100 ng/µl peptide pheromone to ensure induction of gene expression^35^ and harvested three hours later for western blot analysis using an anti-ESAT-6 antibody for detection. Figure 2 shows that AgE6 was present in protein extracts of all strains expected to produce the antigen, with major bands appearing at expected positions. The expected sizes of the fusion proteins vary since the lengths of the anchor sequences differ. Additional bands likely result from proteolytic degradation of the target protein which is to be expected if the cells are stressed and which is commonly observed in experiments like these^33,38^. The strength of the bands varied between strains and anchors, indicating variation in production levels.

**Figure 3:**
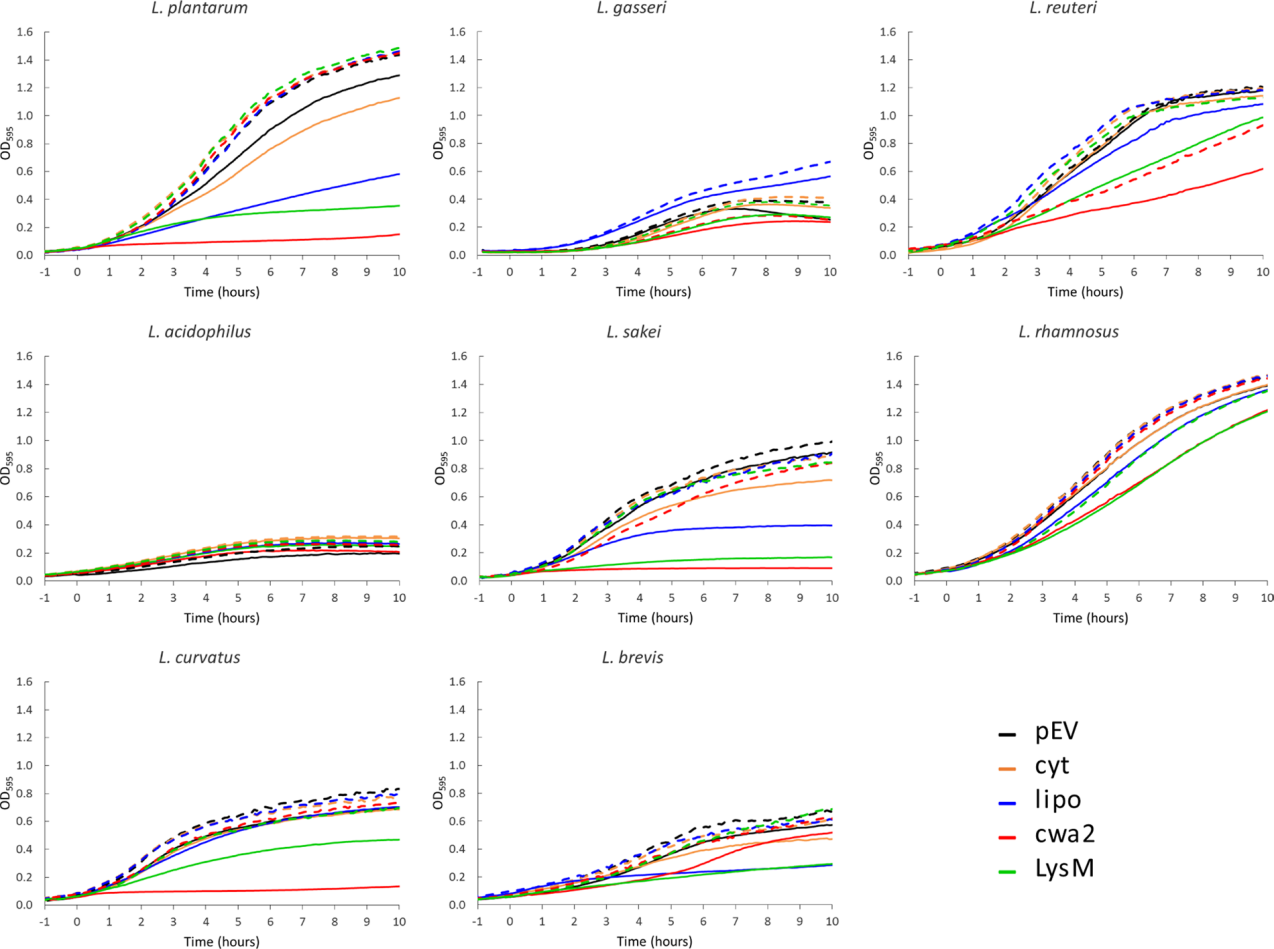
Growth of the recombinant *Lactobacillus* strains. The growth curves are for lactobacilli harbouring plasmids for expression of intracellular (cyt) or surface-displayed (lipo, cwa2, LysM) AgE6-DC and a strain harbouring the empty vector (pEV), with (solid lines) or without (dashed lines) induction of gene expression. Overnight cultures were diluted to an OD_600_ of ~0.02, indicated at −1 hour; at t = 0 the cells were induced by adding the SppIP peptide to a final concentration of 100 ng/µl (solid lines). *L*. *brevis, L*. *sakei* and *L*. *curvatus* were grown at 30 °C whereas the other species were grown at 37 °C. The data used to generate these curves are average of triplicates.

To examine the effect of overproduction of a secreted heterologous protein on the host we measured the growth of the recombinant strains. Figure 3 shows that production of the secreted protein generally reduced growth of the producer strain. Since intracellular production of AgE6 hardly reduced growth rates, it is likely that the adverse effects on growth primarily relate to overloading of the translocation machinery, which may induce stress responses that lead to retarded growth and protein degradation^38^. This is especially noticeable for some of the recombinant *L*. *plantarum, L*. *sakei* and *L*. *curvatus* strains, where induction led to strongly impaired growth.

**Figure 4:**
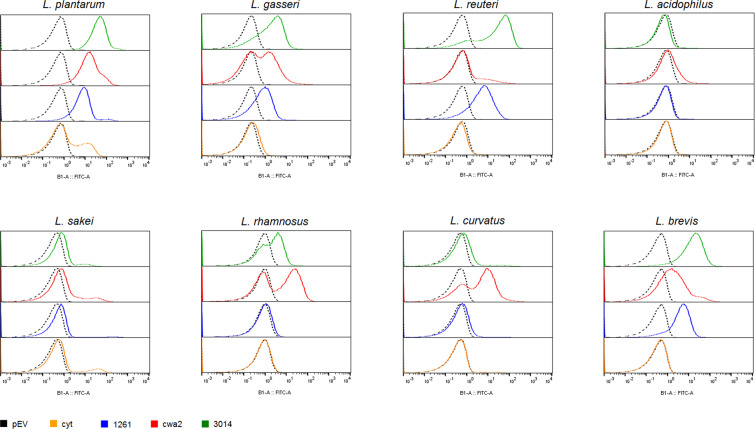
Flow cytometry analysis of surface display of Ag85B-ESAT6-DC(AgE6-DC) in eight species of *Lactobacillus*. Cells were harvested 3 hours after induction with 100 ng/µl of SppIP. pEV, strains harboring empty vector (dotted black lines); cyt, strains harboring a vector for intracellular expression (yellow); lipo (blue), cwa2 (red) and LysM (green), strains harboring various plasmids for expression of surface-anchored antigen. The results for “lipo” have been published previously, where they were reported as MFI, i.e., medians of fluorescence intensity^30^. The data presented are from one representative experiment, out of at least three experiments in total.

**Figure 5:**
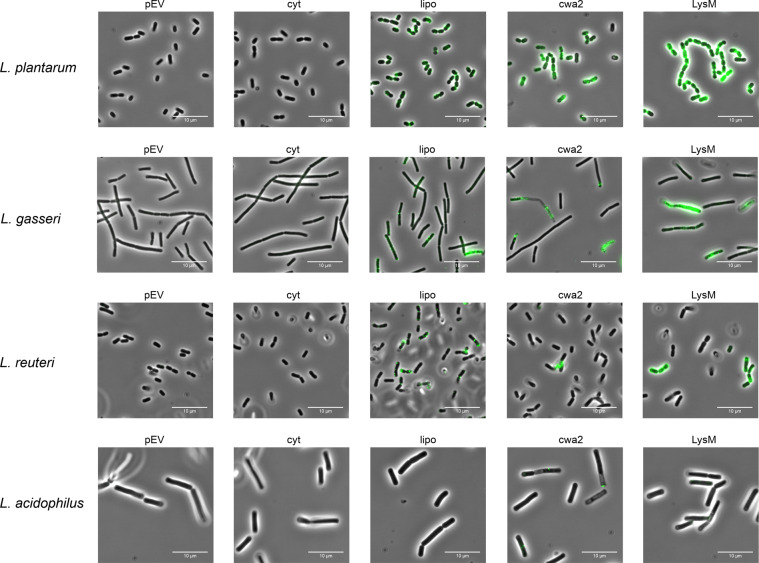
Analysis of surface display of Ag85B-ESAT6-DC(AgE6-DC) using indirect immunofluorescence microscopy. Cells were harvested 3 hours after induction with 100 ng/µl of SppIP. pEV, strains harboring empty vector; cyt, strains harboring the vector for intracellular expression; lipo, cwa2 and LysM, strains harboring various plasmids for expression of surface-anchored antigen. Parts of the “lipo” pictures for *L*. *plantarum* and *L*. *reuteri* and have been published previously^30^. The data presented are from one representative experiment, out of at least three experiments in total.

**Figure 6:**
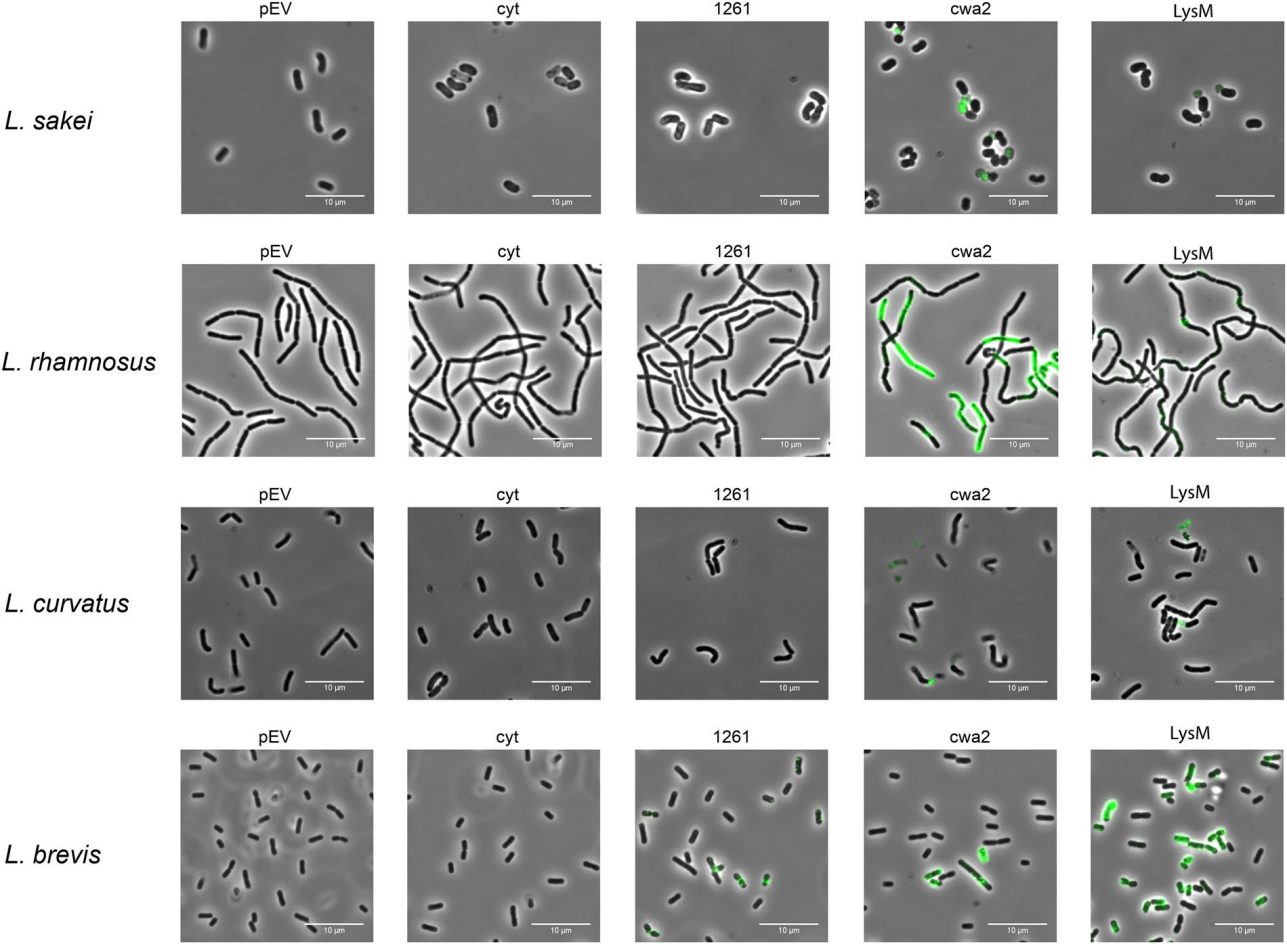
Analysis of surface display of Ag85B-ESAT6-DC(AgE6-DC) using indirect immunofluorescence microscopy. Cells were harvested 3 hours after induction with 100 ng/µl of SppIP. pEV, strains harboring empty vector; cyt, strains harboring the vector for intracellular expression; lipo, cwa2 and LysM, strains harboring various plasmids for expression of surface-anchored antigen. Part of the “lipo” picture for *L*. *brevis* has been published previously^30^. The data presented are from one representative experiment, out of at least three experiments in total.

To scrutinize surface localization of the AgE6 anchored proteins we used flow cytometry, which showed a clear increase of the fluorescence signal for most of the bacteria in which AgE6 was expected to be anchored to the cell wall via LPXTG and LysM motifs, compared to the negative control (pEV; Fig. 4). No surface display was detected for *L*. *acidophilus*, although the western blot analysis (Fig. 2) confirmed production of both hybrid proteins. One possible explanation is that the Lp_3050 signal peptide, derived from *L*. *plantarum*, is not functional in *L*. *acidophilus*. It is well known that the secretion efficiency of heterologous proteins can be highly affected by the choice of signal peptide^33,39^. Fluorescence microscopy analysis confirmed surface exposure of cell wall-anchored AgE6 for seven of the recombinant strains (Figs. 5 and 6). Of note, signal intensities did differ between positive strains and between the two anchors, which may be due to different levels of secretion and anchoring and/or to differences in cell wall architecture that may lead to differences in accessibility for the antibodies used for detection. *L*. *plantarum*, the source of the LPXTG- and LysM-based anchoring sequences, showed strong signals in flow cytometry and fluorescence microscopy (Figs. 4 and 5), which suggests that the use of homologous secretion and anchoring signals is beneficial.

As described previously^30^, strains with lipo-anchored AgE6 gave more varying results. Both flow cytometry and fluorescence microscopy showed no or only very weak signals for *L*. *acidophilus, L*. *sakei, L*. *rhamnosus* and *L*. *curvatus*, whereas clear signals were obtained for the four other strains (Figs. 4–6). These variations may indicate differences in the amounts of surface-displayed protein, but may also reflect differences in the actual exposure of the antigen. Bacteria show large variation in the composition, thickness and porosity of the peptidoglycan layer and such variations may greatly affect the degree of surface exposure and prominence of a membrane anchored target protein^40^; Fig. 1). Interestingly, the microscopic analyses displayed in Figs. 5 and 6 show weaker signals for membrane-anchored antigen, compared to cell wall-anchored antigen, for all *Lactobacillus* strains. This supports the idea that cell wall-anchored antigen is more accessible for antibody detection. Of note, our previous work has shown that *L*. *brevis, L*. *gasseri, L*. *reuteri* and *L*. *plantarum* expressing the lipo-anchored antigen vary in terms of their potential to induce cellular and humoral immunity^30^.

It is worth noting that both the flow cytometry data (Fig. 4) and the immune fluorescence microscopy (Figs. 5 and 6) show that there is heterogeneity within the populations of antigen-displaying cells. The flow cytometry data sometimes show bimodality, indicating the existence of two populations of the bacteria (Fig. 4), in particular for the “cwa” strains, i.e., those expressing the sortase-anchored antigen. Likewise, the microscopy shows mixtures of coloured and non-coloured cells. While these latter differences are partly due to the fact that not all cells are fully in focus, some differences seem genuine. For example, *L*. *gasseri* expressing cell-wall anchored antigen shows a strong bi-modality in flow cytometri (Fig. 4), while the microscopy convincingly shows the existing of populations of coloured and non-coloured cells (Fig. 5, “cwa2” sample). It is possible that these heterogeneities reflect differences in the cellular growth phase, which may affect the amount or “visibility” (i.e., accessibility for the antibody) of the displayed antigen. We were not able to detect clear correlations between the observed heterogeneity and other features, such as the growth curves displayed in Fig. 3.

Interestingly, the fluorescence microscopy also shows heterogenicity of the signal within the cells, indicating that the antigen is not evenly distributed. This is especially pronounced for cells expressing antigens with lipoprotein and LPXTG anchors. As for the LPXTG-anchors, it has been shown that there is a close connection between the regulation of cell division and protein anchoring. Some LPXTG-anchored proteins rapidly appear and accumulate at the septum during bacterial growth, whereas others gather at the poles, where the cell wall is older^41^. The observed heterogeneity could thus be explained by the combination of (unknown) cellular localization signals in the employed signal peptide or anchoring sequence and variation in the cellular growth phase.

While the present study shows that anchoring signals from *L*. *plantarum* function in multiple *Lactobacillus* species, there are obvious opportunities for further optimization, for example using homologous anchoring signals. In this respect, it is worth noting that there is considerable species-specific variation in the LPXTG motif that directs sortase-mediated anchoring in lactobacilli^42,43^. While several lactobacilli do show the LPXTG consensus sequence, with x often being a Q, the consensus motif for *Lactobacillus plantarum* is LPQTXE^44^ and the motif used in this study was LPQTSE. This specific anchoring motif may not be equally well compatible with the sortase systems of all tested *Lactobacillus* species. This could explain part of the variation in the growth characteristics of the various strains expressing cell-wall anchored antigen, and may also be a factor underlying variation in the amount. Variations in the functionality of the sortase system could possibly also help explaining the observed variation in the distribution of the surface-displayed antigen between and within cells.

In conclusion, the present study shows that surface anchors derived from *L*. *plantarum* WCFS1 are promising candidates for anchoring of heterologous proteins in other *Lactobacillus* species. The various recombinant strains, containing various anchors, showed varying efficacies in terms the apparent amounts of surface-displayed antigens. The strains also varied in terms of how production of the antigen affected growth and here covalent anchoring encoded by pCwa2 led to the largest growth inhibition. All in all, however, the present study shows that the AgE6 hybrid mycobacterial antigen could be displayed on the surface of seven of the eight tested lactobacilli, with at least one of the three tested anchors. In the case of *L*. *plantarum*, *L*. *gasseri*, *L*. *reuteri* and *L*. *brevis*, all three anchoring strategies worked.

In the pSIP plasmids, fragments encoding promoters, target proteins, signal peptides and anchoring sequences are separated by linkers with restriction sites, allowing easy exchange of the different parts. Considering that it is difficult to predict the success of vector designs, this cassette-like structure, which allows easy and fast screening of multiple set-ups, is beneficial.

## Materials and Methods

### Bacterial strains and growth conditions

Bacterial strains used in this study are listed in Table 1. *Lactococcus lactis* was grown in M17 broth (Oxoid, Hampshire, Uk) supplemented with 0.5% (w/v) glucose at 30 °C without agitation. Lactobacilli were grown in MRS broth (Oxoid) without agitation. *L*. *plantarum, L*. *gasseri, L*. *reuteri, L*. *acidophilus* and *L*. *rhamnosus* were grown at 37 °C; *L*. *sakei, L*. *curvatus* and *L*. *brevis* were grown at 30 °C.

When appropriate, erythromycin was used at a concentration of 10 µg ml^−1^ for *Lactobacillus* and *Lactococcus*, and 200 µg ml^−1^ for *Escherichia coli*, both in broth and solid media.

### Plasmid construction and DNA manipulation

Previously developed expression vectors (Table 1) were modified by exchanging the narrow range replicon (256_rep_) with the broad range SH71_rep_ replicon. The plasmid Lp_1261AE6-DC_SH71^30^, here referred to as pLipo, was digested with *Bgl*II and *Hin*dIII and the 5.6 kb fragment containing SH71_rep_ was ligated to a 2.2 kb fragment of pLp_3050AgE6cwa2^9^ generated by using the same restriction enzymes, yielding pLp_3050DC_AgE6cwa2_SH71, referred to as pCwa2.

pLp_3014AgE6-DC_SH71 was constructed by amplifying the AgE6-DC hybrid antigen from pLp_1261AgE6-DC^9^ using primer pair Ag85Fus3014F/Ag85DC-R (Table 2), after which the resulting 1.2 kb PCR fragment was digested with *Sal*I/*Eco*RI and inserted into the pLp_3014Inv^18^ vector digested with the same enzymes. Subsequently, the 256_rep_ replicon in the constructed plasmid was exchanged with the SH71 replicon from Lp_1261AgE6-DC_SH71 using *Bgl*II and *Hin*dIII, yielding pLp_3014AgE6-DC_SH71, referred to as pLysM.

**Table 2:** Primers used in this study.

Primer	Sequence(5′→3′)*	Description
pNdeISIP_F	GGAGTATGATT*CATATG*TTTAGTCGTCCAGGTTTGC	Forward primer for amplification of AgE6 from pUC57-AgE6
pAgESATCyt-R	GGAAACAGCTATGACCATGATTAC	Reverse primer for amplification of AgE6 from pUC57-AgE6
Ag85Fus3014F	CAACGAGTTCAACT*GTCGAC*TTTAGTCGTCCAGGTT	Forward primer for amplification of AgE6 from Lp_1261AgE6-DC. Contains *Sal*I restriction site.
Ag85DC-R	GCCAAGCTTC*GAATTC*TTATGGCCGTTGTGGCGT	Reverse primer for amplification of AgE6 from Lp_1261AgE6-DC. Contains an *Eco*RI restriction site.

*Restriction sites in italics.

The plasmid for intracellular production of AgE6-DC was made by amplifying the AgE6 fragment using the primer pair pNdeISIP_F and pAgESATCyt-R (Table 2) using pUC57-AgE6 (Genscript) as template. The resulting PCR product was digested with *Nde*I/*Acc*65I and ligated into the same restriction sites of pLp_1261AgE6-DC^9^, yielding pLp_cyt:AgE6-DC. The 256_rep_ replicon in pLp_cyt:AgE6-DC was exchanged with the SH71 replicon from Lp_1261AgE6-DC_SH71 using the restriction enzymes *Age*I and *Hin*dIII, resulting in pLp_cyt:AgE6-DC_SH71, referred to as pCyt.

Plasmids harboring the 256_rep_ replicon were propagated in *E*. *coli*, while SH71 containing plasmids was propagated in *L*. *lactis*, before transformation to competent *Lactobacillus* species (Table 1). From *E*. *coli*, all plasmids were isolated by using the Plasmid Nucleospin miniprep kit from Macherey-Nagel, following the manufacturer’s protocol. For isolation of plasmids from *L*. *lactis*, the cells were pretreated with lysozyme (10 mg ml^−1^) and mutanolysin (100 U ml^−1^) for 30 min at 37 °C, before proceeding with the lysis step in the Plasmid Nucleospin miniprep protocol. All PCR amplified sequences were verified by DNA sequencing.

### Preparation of competent cells and electroporation

*Lactobacillus* strains were made electro-competent and transformed as described in^35,45^, except for *L*. *reuteri*. For transformation of *L*. *reuteri*, an overnight culture was diluted in 50 ml fresh pre-warmed MRS to an OD_600_ of 0.1, after which the culture was grown until an OD_600_ of 0.7 at 37 °C. The cells were harvested and washed twice in ice cold electroporation buffer (0.5 M sucrose, 10% (w/v) glycerol) after which the cell pellet was resuspended in 800 µl electroporation buffer and divided into aliquots of 40 µl. Forty microliters of the cell suspension and 0.1 to 2.5 µg of plasmid DNA were mixed and added to a 0.2-cm cuvette for each transformation. Pulses were applied with settings of 2.5 kV, 200 Ω, and 25 µF (Gene Pulser and Pulse Controller; Bio-Rad Laboratories, Richmond, CA).

### Induction of gene expression and harvesting of recombinant Lactobacilli

Overnight cultures of lactobacilli were diluted in MRS with 10 µg ml^−1^ erythromycin to an OD_600_ of 0.15, followed by incubation at the appropriate temperature without agitation. When the OD_600_ reached ~0.3 the cultures were induced by adding 100 ng ml^−1^ SppIP (Caslo ApS, Lyngby, Denmark^46^). The cultures were harvested 3 hours after induction by centrifugation at 5 000 × g for 5–10 min. The cells were washed once in PBS and stored at −20 °C, before proceeding with SDS-PAGE or the staining procedure.

### Western blots

Cells from 25 ml of harvested culture were disrupted with glass beads (size <106 µm, Sigma -Aldrich, St. Louis, MI) using a FastPrep-24 instrument (MP Biomedicals, Santa Ana, CA) using 6.5 m/s speed for 45 second, three times. The protein extracts, in amounts that were adjusted based on the OD_600_ at harvesting, were applied to a Mini-PROTEAN TGX Stain-Free Gel (BioRad, Hercules, CA) using Tris/Glycine/SDS as a running buffer. The proteins on the gel were blotted to a nitrocellulose membrane using an iBlot Dry Blotting System (Invitrogen, Carlsbad, CA) and antibody hybridization was performed using SNAP i.d. 2.0 (Millipore, Burlington, MA) following the instructions of the manufacturer.

Antibodies were used at the following concentrations: monoclonal mouse anti-ESAT-6 (Abcam, Cambridge, UK, ab26246) diluted 1:15 000; polyclonal horseradish peroxidase (HRP) rabbit anti-mouse IgG (Dako, Santa Clara, CA) diluted 1:4 000–1:6 000. SuperSignal West Pico PLUS Chemiluminescent Substrate (Thermo, Waltham, MA) was used to visualize protein bands following the protocol provided by the manufacturer.

### Flow cytometry and microscopy

Bacterial cultures were grown and induced as described above. Cells from approximately 0.5 ml of culture were harvested and washed once with PBS. The bacteria were resuspended in PBS with 2% (w/v) BSA and incubated for 30 min at room temperature with monoclonal mouse anti-ESAT-6 (Abcam, ab26246) diluted 1:250. After washing the cells three times with PBS containing 2% (w/v) BSA, they were incubated for 30 min with anti-mouse IgG-FITC antibody (F9137, Sigma -Aldrich), diluted 1:166. After repeating the washing step, the bacteria were analyzed using a MACSQuant analyzer (Miltenyi Biotec GmbH, Bergisch Gladbach, Germany) following the manufacturer’s protocol. Additionally, the stained bacteria were visualized by immunofluorescence microscopy using an Axio Observer.z1 microscope (Zeiss, Oberkochen, Germany) using excitation wavelengths of 450 to 490 nm and emission wavelengths of 500 to 590 nm.

## Data Availability

All the data used in the present study are provided within the main manuscript.
